# The Effects of Varying Intensities of Unilateral Handgrip Fatigue on Bilateral Movement

**DOI:** 10.3390/brainsci16010047

**Published:** 2025-12-29

**Authors:** Adrian L. Knorz, Justin W. Andrushko, Sebastian Sporn, Charlotte J. Stagg, Catharina Zich

**Affiliations:** 1 Centre for Integrative Neuroimaging, FMRIB, Nuffield Department of Clinical Neurosciences, University of Oxford, Oxford, UK; charlotte.stagg@ndcn.ox.ac.uk; 2 School of Sport, Exercise and Rehabilitation, Faculty of Health and Wellbeing, Northumbria University, Newcastle upon Tyne, UK; justin.andrushko@northumbria.ac.uk; 3 Department of Clinical and Movement Neurosciences, UCL Queen Square Institute of Neurology, London, UK; s.sporn@ucl.ac.uk; 4 Medical Research Council Brain Network Dynamics Unit, Nuffield Department of Clinical Neurosciences, University of Oxford, Oxford, UK

**Keywords:** contralateral adaptation, kinematic assessment, motor control, motor learning, muscle fatigue

## Abstract

**Background/Objectives**: The ability to maintain movement quality despite muscle fatigue is essential for daily activities and preserving independence after motor impairments. Many real-life situations involve asymmetrical muscle activation, leading to unilateral muscle fatigue. Repeated unilateral handgrip contractions at submaximal force have been linked to neural changes in both contralateral and ipsilateral motor areas, as well as improved contralateral response times in a button-press task. However, it remains unclear whether these improvements in response latency extend to higher-level benefits in overall arm movement quality. **Methods**: Thirty healthy participants performed unilateral handgrip fatiguing tasks at 5%, 50%, and 75% of maximum voluntary contraction (MVC) force. Subsequently, bilateral upper-limb movement quality was assessed in an object-hit task using a Kinarm robot. **Results**: The 50% and 75% MVC protocols elicited muscle fatigue as evidenced by declines in force output, post-exercise MVC, electromyography magnitude changes, and increased perceived exertion compared to the 5% MVC control condition. However, no significant changes in kinematic measures of the object-hit task were observed for either the fatigued (ipsilateral) or non-fatigued (contralateral) arm, indicating that unilateral handgrip fatigue did not affect higher-level movement quality. **Conclusions**: Previously reported improvements on contralateral response latency in a button-press task were not found to translate into advanced arm movement quality benefits.

## 1. Introduction

The human body’s ability to adapt to muscle fatigue is crucial for a wide range of daily activities. In healthy individuals, this adaptability enables accurate movements despite fatigue, benefiting athletic performance and physically demanding professions. For patients with motor impairments, maintaining movement quality despite fatigue is essential for preserving independence and reducing reliance on external care. Muscle fatigue is a complex, often vaguely defined state resulting from various peripheral and central processes. While it can sometimes be broadly defined as a ‘transient decrease in the capacity to perform physical actions’ [1], it is often more precisely characterised as an ‘exercise-induced decrease in the ability to produce force’ [2].

Muscle fatigue can be quantified by various physiological markers, such as a decline in force output and distinctive electromyographical changes [1]. Muscle fatigue typically develops gradually, and its physiological effects are often task-dependent. While fatigue can occur symmetrically, real-life situations are rarely perfectly symmetrical, with healthy individuals often facing asymmetric stresses on their motor system. For example, one might grip a bus handle with one hand for an extended period, then perform a more complex task with the contralateral, unfatigued arm, such as inserting a key or engaging in bimanual actions like taking off a coat and untying their shoes. How preceding unimanual fatigue will affect subsequent movement quality, whether in the contralateral or bilateral limbs, depends on changes in neural activity triggered by unimanual fatigue, which can influence both the fatigued limb and the unfatigued homologue. Recent evidence from our research suggests that repeated unilateral handgrip contractions at 50% of maximum voluntary contraction (MVC), which decrease volitional force output, are linked to improved motor response latency in the contralateral, unfatigued limb in a button-press task compared to a 5% MVC control condition [3].

Functional magnetic resonance imaging (fMRI) and magnetic resonance spectroscopic imaging (MRSI) revealed that changes in gamma-aminobutyric acid (GABA) levels in the ipsilateral primary motor cortex (M1) and bilateral supplementary motor areas (SMA) were related to improvements in contralateral response time [3]. GABA drives cortical inhibition, and a reduction in GABA-related activity in M1 promotes plasticity and motor learning [4]. This could explain why decreased GABA in the ipsilateral M1 and bilateral SMAs after unimanual contractions correlates with improved button-press response time [3]. Another key neural effect is the increased connectivity between the SMAs of both hemispheres following unimanual fatigue [3]. Since callosal fibres primarily mediate inhibition [5], this increased SMA–SMA connectivity likely reflects enhanced interhemispheric inhibition (IHI). While this might seem contradictory to the disinhibition in M1 and SMA, these mechanisms may work together to improve response time. Increased IHI through SMA–SMA connectivity could focalise disinhibition in the opposite hemisphere, enabling a more focused “crossed-facilitation” effect. Callosal fibres, which are primarily excitatory, can facilitate their target areas in the opposite hemisphere, with inhibition occurring through interactions with local inhibitory cells [5]. The result would then be a ‘focal interhemispheric facilitation’, with IHI limiting the spread of excitation. This raises the question of how far the facilitatory effects extend from the target area and whether they could have broader effects on motor behaviour than seen in our previous experiment.

It remains unclear which range of fatigue intensities (i.e., percentages of MVC) can trigger neural responses, as previous experiments typically used a single fatigue intensity (e.g., 50% of MVC vs. 5% of MVC as a control). Although we have shown that a decline in GABA following unilateral handgrip contractions correlates with improved contralateral response time, it is unclear whether this translates into quantifiable benefits in higher-level movement quality. This is particularly relevant when considering motor skills, which may not only require speed but also accuracy. Branscheidt et al. have even suggested that muscle fatigue can lead to longer-term target overshoot due to excessive force production [6]. These seemingly contradictory effects of muscle fatigue may reflect a trade-off: the disinhibitory effects of reduced GABA might enhance speed and force but at the cost of accuracy, leading to potential target overshoot.

Previous research has highlighted potentially conflicting effects in the supraspinal response to unilateral muscle fatigue. Studies on unilateral fatigue in the right dorsal interosseus [7] and knee extensors [8] reported varying degrees of cross-over effects on the contralateral homologue, while a study on right unimanual fatigue found no such effect [9]. A transcranial magnetic stimulation (TMS) study on unilateral handgrip contractions showed decreased short interval intracortical inhibition (SICI) in the ipsilateral M1, suggesting reduced excitability [10]. In contrast, other studies have found that forceful exercise of the left abductor pollicis brevis (APB) at >50% MVC can produce anti-inhibitory effects on the contralateral homologue [11]. These inconsistencies are likely due to the heterogeneity of experimental protocols.

Cross-over effects of fatigue have typically been studied using contralateral tasks specific to the fatigued muscle group. However, growing evidence suggests that fatigue can also affect motor pathways of task-unrelated contralateral muscle groups. For example, sustained handgrip contractions have been shown to alter motor-evoked potentials (MEPs) in both ipsilateral and contralateral knee extensors [12], while fatiguing the dominant knee extensors affects the excitability of the non-dominant biceps brachii corticomotor pathway [13]. These findings suggest that the neural effects of fatigue extend beyond task-specific contralateral homologues. This raises the possibility that such changes in neural activity could be leveraged to support motor learning. Our group has previously demonstrated improved contralateral button-press response time after unilateral handgrip contractions [3], suggesting that changes in functional connectivity and GABA-related signalling could potentially affect contralateral motor behaviour more broadly than initially tested, for example, affecting movement quality of the entire arm rather than merely finger movement velocity.

Inferring the movement quality of bilateral compound movements from unilateral measurements is challenging. As Swinnen [14] noted, ‘principles of interlimb coordination are unique and cannot be inferred from the laws of single limb movements’. Feeney et al. [15] further emphasised the importance of coordinated interlimb movements in a fatigued state, suggesting that ‘switching to bimanual tasks when one hand becomes fatigued could be beneficial regarding preserving the high level of both the manipulation performance and force coordination’.

This study aimed to induce varying intensities of unilateral handgrip fatigue and assess its impact on movement quality through advanced kinematic parameters of both arms during bilateral tasks. We hypothesised that previously observed task-specific benefits in contralateral button-press response time would extend to overall arm movement quality in an object-hit task, with improvements scaling linearly with increasing ipsilateral fatiguing intensity. Additionally, we expected that unimanual fatigue would impair the ipsilateral arm performance during the bilateral task, with compensation from the contralateral arm, also scaling with fatigue intensity. We designed our experiment to require both speed and fine motor accuracy, providing leverage to identify issues with overshoot and rapid bimanual coordination. Additionally, the assessment task engaged not only handgrip muscles but also upper arm muscles, which map to adjacent cortical areas. This design enabled us to explore whether unilateral handgrip fatigue affects broader movement quality in the arm.

## 2. Materials and Methods

### 2.1. Participants

We recruited 30 right-handed participants, between 19 and 41 years of age (median: 25, IQR: 10.75), of which 18 (60%) were female. Informed consent was acquired in written form from all participants. This study has been approved by the Oxford Central University Research Ethics Committee (CUREC) (R88022/RE003) and was performed in accordance with the Declaration of Helsinki. Participants reported no current psychiatric or neurological disorders and had normal or corrected-to-normal vision. While a formal a priori power analysis was not conducted, the sample size in our study is comparable to previous research using similar fatigue protocols [3,8], which have reliably detected effects of fatigue on performance measures.

### 2.2. Session Overview

Each participant completed a single session (~2.5 h duration). All instructions and encouragements provided to participants followed a detailed script, ensuring consistency across participants.

#### 2.2.1. Acclimatisation and Pre-Assessment

After informed consent, participants were first allowed to become accustomed to the Kinarm robot. The chair height was adjusted to enable comfortable operation of the robotic handles, while the position of the chair was kept strictly centred relative to the Kinarm robot. Participants underwent 1 min of training to familiarise themselves with the Assessment Task (AT).

To individualise the force levels for the Fatiguing Task (FT), each participant’s maximum voluntary contraction (MVC) was quantified. Participants performed 3 cued contractions at MVC, each of 3 s durations with 30 s of rest in between contractions (MVC 3×). Verbal encouragement was given during MVC attempts to facilitate maximum efforts and force output. By employing the 2 × 2 Achievement Goals Questionnaire for Sport (AGQ-S) by Elliot and McGregor [16] directly before the MVC calibration, we aimed to further encourage participants and to quantify their performance motivation.

After quantifying individual MVC (via MVC 3×), participants completed several questionnaires: Edinburgh Handedness Inventory (EHI) [17], World Health Organization Global Physical Activity Questionnaire (GPAQ) [18], and BORG-CR-10 [19]. Next, a single MVC (i.e., MVC 1×) was obtained, followed by the baseline AT.

#### 2.2.2. Main Experiment

**Figure 1:**
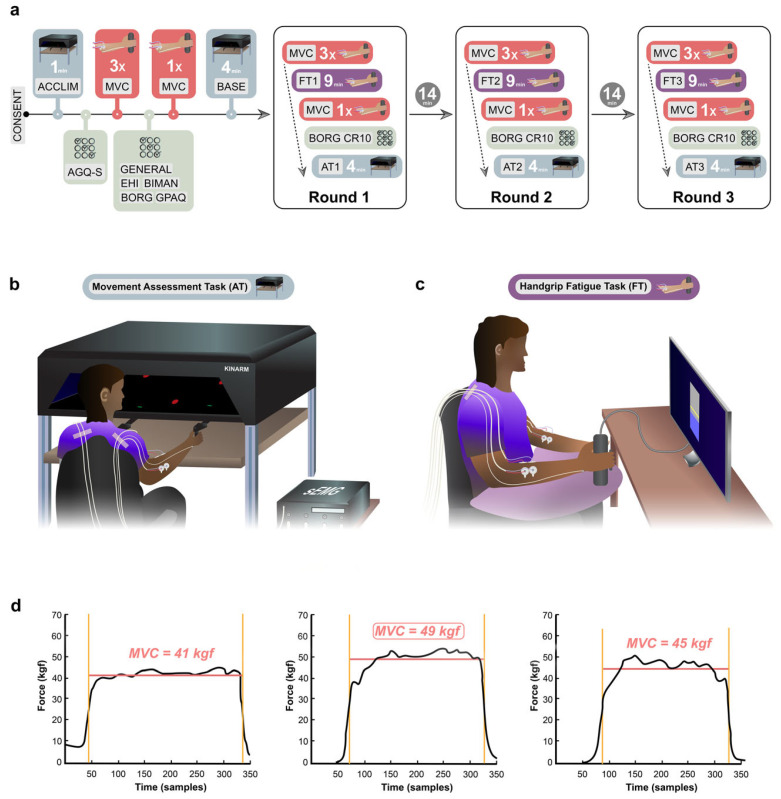
Experimental design. (**a**) Experimental timeline. Abbreviations: CONSENT = Informed consent of participants, ACCLIM = Kinarm acclimatisation period, AGQ-S = Achievement Goals Questionnaire for Sport, MVC = Maximum Voluntary Contraction, GENERAL = General and Demographic Questionnaire, EHI = Edinburgh Handedness Inventory, BIMAN = Bimanual Tasks in Daily Life Questionnaire, BORG = BORG-CR10 rating scale, GPAQ = WHO Global Physical Activity Questionnaire, BASE = Kinarm baseline period. (**b**) Experimental setup of the movement Assessment Task (AT). (**c**) Experimental setup of the handgrip fatigue task (FT). (**d**) Example of calculation of MVC3.

During the main experiment, participants performed three task rounds of the following: MVC 3×, Fatiguing Task (FT), MVC 1×, BORG-CR10, Assessment Task (AT), and a recovery period (Figure 1a). The only difference between the three rounds was the level of force intensity (5%, 50%, or 75% of MVC) during the FT.

#### 2.2.3. Fatiguing Task (FT)

We employed a visually prompted handgrip FT of 9 min duration, consisting of 1 s handgrip contractions followed by 1 s relaxation periods for a total of 270 contractions, as shown previously [3]. Similar fatiguing task protocols using repeated handgrip contractions have been shown to effectively elicit both central effects on neural activity [20] and peripheral effects of fatigue [21]. Participants were instructed to adjust their applied handgrip force to match a target line displayed on a computer screen (Figure 1c). Participants were shown their handgrip force output in real time. The horizontal target line was kept in the centre of the screen to maintain a consistent visual task presentation across the three conditions, regardless of the force intensity required (i.e., force intensity of 5%, 50%, or 75% of baseline MVC).

The three force intensity conditions (5%, 50%, 75% MVC) were fully counterbalanced across participants. To this end, six experimental protocols (5, 50, 75; 5, 75, 50; 50, 75, 5; 50, 5, 75; 75, 5, 50; 75, 50, 5). Each protocol comprised five subjects. Participants were allocated to a protocol using covariate-adaptive allocation, employing a variance minimisation procedure developed by Sella et al. [22]. Thus, participants were assigned using a covariate-adaptive allocation procedure that balanced groups on key participant characteristics (e.g., MVC values). This variance minimisation method adjusts each new participant to reduce differences between protocols while retaining an element of randomisation.

#### 2.2.4. Assessment Task (AT)

To assess the effect of different force intensities of unimanual fatigue on the movement quality of both arms, participants completed a bimanual object-hit task for a duration of 4 min. The task was adapted from the original object-hit task by Tyryshkin et al. [23], which reliably demonstrated sensitivity to detect motor impairment, with ‘effectively no floor or ceiling effects’ [23]. This is further supported by another study, which states that ‘even participants with mild sensorimotor deficit according to clinical classification (MACS I) were impaired on this task’ [24]. To complete the task, participants needed to perform arm reaches with robotic handles (displayed to the participant as virtual green paddles of 1.5 cm width and 0.5 cm height) to deflect virtual red balls (0.75 cm diameter) away (Figure 1b). Balls were continuously released from 16 evenly distributed, non-overlapping bins at the rear/top side of the display (30 objects per bin), falling vertically toward the participant’s side of the display at different velocities (15–30 cm/s) with two balls per second. The bin and speed of the falling virtual objects were kept continuously pseudorandom and unpredictable to the participant. The starting location of the handles was in a neutral and comfortable position at the centre of the left and right workspace (76 cm × 44 cm), respectively. To enable detection of assistive spillover movements, i.e., possible attempts by the unfatigued arm to assist on the contralateral side of the workspace, both handles were allowed to cross the midline. This also implied that they could technically bump into each other. The comparably small width of the paddles was chosen to increase the accuracy of movements required to hit a ball. When the participant hit a ball with the paddle, the ball’s trajectory deviated in a physically plausible direction after collision, providing real-time feedback to the participant.

#### 2.2.5. Recovery Period

The recovery period between task rounds was 20 min, which has been shown to be adequate to allow for good restitution of most elements of voluntary muscle force activation [25]. During the first recovery period after the first experimental round, participants were shown a standardised 14 min section from a documentary movie (‘Planet Earth’, S01). During the second recovery period after the second experimental round, participants then watched the subsequent 14 min section from the same movie.

### 2.3. Data Acquisition

#### 2.3.1. Force Data Acquisition

MVC and FT were conducted using a hand dynamometer (Biopac Systems Inc., Aero Camino Goleta, CA, USA) and custom code implemented in MATLAB R2022a (The MathWorks Inc., Natick, MA, USA) using the Psychophysics Toolbox extensions (Psychtoolbox-3) [26,27]. Force data were sampled at 47.75 Hz. Dynamometer force data were routed through Lab Streaming Layer (LSL) and LabRecorder 1.16.0 (https://github.com/labstreaminglayer/App-LabRecorder/releases/tag/v1.16.0).

#### 2.3.2. Kinematic Data Acquisition

Kinematic movement quality assessments were acquired using a Kinarm End-Point Lab robot with Dexterit-E 3.9.2 software (BKIN Technologies Ltd., Kingston, ON, Canada).

#### 2.3.3. EMG Data Acquisition

During performance of MVC, FT, and AT, surface electromyography (EMG) of the right and left flexor carpi radialis (FCR) and extensor carpi radialis longus (ECR) muscles was acquired using a bipolar configuration with a TMSi Porti7 recorder (Twente Medical Systems International B.V., Oldenzaal, The Netherlands). Small adhesive disposable electrodes with a contact area of 4.5 cm^2^ (Kendall H124SG; Cardinal Health, Dublin, OH, USA) were used and placed in a bipolar setup with two electrodes placed next to each other on the bulk of the muscle belly at a fixed distance of 24 mm between the centres of the electrodes. Participants were instructed to perform a handgrip contraction during which the muscles were palpated manually to determine correct placement. A common grounding electrode was placed on the ulnar styloid process. EMG signals were recorded in raw format at a sampling rate of 2048 Hz and were routed through LSL and LabRecorder.

### 2.4. Data Analysis

#### 2.4.1. MVC Data

Median force was calculated for each MVC separately by calculating the median force applied between the full width at half maximum (FWHM) points of each contraction force profile (Figure 1d). For MVC 3×, the highest of the 3 median values of the 3 MVCs from each participant was used for statistical analysis. The highest median value of the MVC 3× at baseline was used as a reference value for computing subsequent target values for the FT.

#### 2.4.2. Force Data

The first 10 of the 270 separate contractions of each FT were classed as a familiarisation period and hence excluded from the analysis. For the remaining 260 contractions, the area under the curve (AUC) was computed over the 1 s contraction window for each contraction, as shown before [3]. Next, the slope of the 260 AUC values was computed using linear regression. The derived beta value of the regression was subsequently utilised for force output quantification, with a negative beta value indicating declining force output, which represents one of the main criteria for identifying muscle fatigue.

#### 2.4.3. Kinarm Data

From the kinematic data, the number of hits per hand was calculated as the primary outcome measure. Secondary outcome measures comprised left and right hand velocity, (absolute) acceleration, and space covered. Space covered was defined as the area a hand covered across the entire task duration.

#### 2.4.4. EMG Data

Using MATLAB R2022a (The MathWorks Inc., Natick, MA, USA), the EMG data were bandpass filtered between 10 Hz and 500 Hz with a fourth-order Butterworth filter, and the envelope of the EMG data was computed. During the FT, the EMG data were analysed in a similar manner to the Force data, i.e., we removed the first 10 contractions, calculated the AUC per contraction, and then computed a linear regression across contractions. During the AT, we computed the median of the envelope from the beginning to the end of the task.

### 2.5. Statistical Analysis

Statistical analyses were performed using Prism version 10.2.2 (Graphpad, San Diego, CA, USA), JASP version 0.18.3 (University of Amsterdam, Amsterdam, The Netherlands) and MATLAB R2022a (The MathWorks Inc., Natick, MA, USA). Differences between the three different force levels of the FT (5%, 50%, 75%) on FT and AT performance were investigated using repeated-measures Analysis of Variance (RM-ANOVA) and follow-up *t*-tests. Pearson correlations were used to assess relationships between the different fatigue measures, except when assumptions were violated, in which case Spearman correlations were applied (e.g., for the BORG scale). We tested whether the force level of the FT affected the space covered by the right (fatigued) or left (unfatigued) hand via a two-sample *t*-test using cluster-based non-parametric permutations [28]. Cluster-based permutation test was used because it controls for multiple comparisons while accounting for the spatial dependence in the two-dimensional data, allowing detection of statistically significant clusters rather than isolated pointwise differences. Row shuffling (5000 permutations) was used to break the link between data and condition labels, creating a null distribution for testing significance without assuming a specific data distribution. A cluster-forming threshold of *p* = 0.01 was used, and a spatial extent threshold of 1 was set to ensure that any cluster had at least 2 adjacent points exceeding the cluster-forming threshold. Partial eta-squared (η_p_^2^) and Cohen’s d (*d*) effect sizes are reported for RM-ANOVA and *t*-tests, respectively. Data visualisations were generated using Prism version 10.2.2 (Graphpad), MATLAB R2022a (The MathWorks Inc., Natick, MA, USA), Adobe Illustrator CC 2024, and Adobe Photoshop CC 2024 (Adobe Inc., San Jose, CA, USA).

## 3. Results

### 3.1. Force Levels During the Fatiguing Task Affect Several Behavioural and Physiological Outcomes

To test whether the three different FT force levels (5%, 50%, 75% MVC) had the expected differential effects on behavioural and physiological markers of fatigue, we first needed to test whether the MVCs acquired before the FT were comparable across the three rounds. A 1 × 3 RM-ANOVA with a within-subjects factor force level (5%, 50%, 75% MVC) demonstrated no significant main effect of force level on MVC (*F*_2,58_ = 0.17, *p* = 0.841, η_p_^2^ = 0.01), suggesting that the MVC did not vary between conditions (5% MVC: *M* = 57.23, *SD* = 17.94; 50% MVC: *M* = 58.18, *SD* = 18.04; 75%: *M* = 57.06, *SD* = 20.04). To test whether the order in which participants performed the three different FT force levels impacted their FT performance, we added a between-subjects effect of order and saw no significant main effect (*F*_5,24_ = 0.41, *p* = 0.83, η_p_^2^ = 0.08) and no order × force interaction (*F*_10,48_ = 1.68, *p* = 0.113, η_p_^2^ = 0.26). This suggests that the recovery period was long enough between rounds to allow comparison of the fatigue conditions within a single-day design.

#### 3.1.1. Performance on the Fatiguing Task Is Inversely Related to Force Level

**Figure 2:**
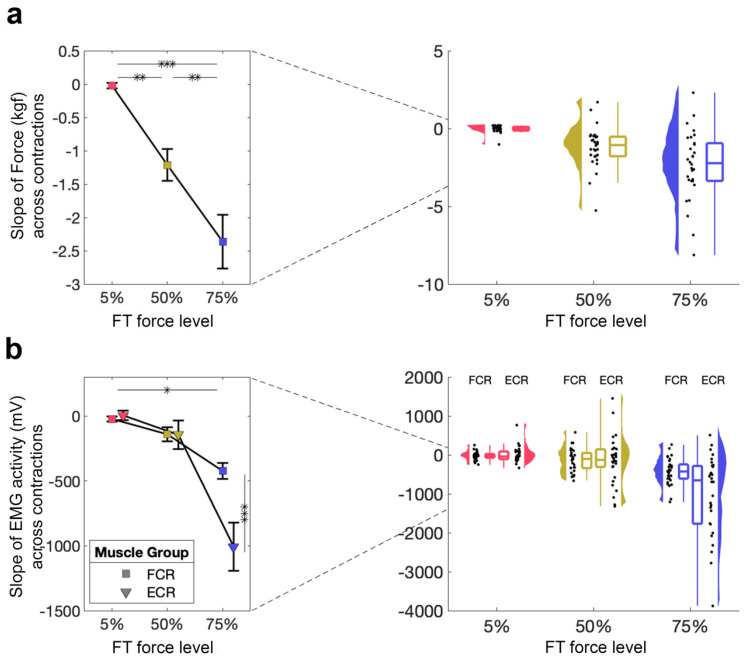
Direct measure of fatigue during the FT. (**a**) FT performance for 5%, 50%, 75%. Mean and standard error across subjects are shown (**left**), as well as single-subject data, distribution, and boxplot (**right**). (**b**) FT muscle activity from the FCR and ECR for 5%, 50%, 75%. Mean and standard error across subjects are shown (**left**), as well as single-subject data, distribution, and boxplot (**right**). * *p* < 0.05, ** *p* < 0.01, *** *p* < 0.001.

We first wanted to examine whether different force levels had different effects on FT performance, as quantified by the slope of the linear regression across the 260 contractions (i.e., AUC values). We tested whether the FT performance was affected by force-level using a 1 × 3 RM-ANOVA with a within-subjects factor of force level (5%, 50%, 75% MVC), which demonstrated a significant main effect of force level (5% MVC: *M* = −0.02, *SD* = 0.23; 50% MVC: *M* = 1.31, *SD* = 1.31; 75% MVC: *M* = −2.36, *SD* = 2.21; *F*_2,58_ = 22.75, *p* < 0.001, η_p_^2^ = 0.44, Figure 2a). All follow-up *t*-tests were significant (5% vs. 50% MVC: *t*_29_ = 3.43, *p*_bonf_ = 0.003, *d* = 0.80; 5% vs. 75% MVC: *t*_29_ = 6.75, *p*_bonf_ < 0.001, *d* = 1.57; 50% vs. 75% MVC: *t*_29_ = 3.32, *p*_bonf_ = 0.005, *d* = 0.77), suggesting that, as expected, the participant’s FT performance deteriorated with increasing force level.

#### 3.1.2. Decrease in EMG Activity During the Fatiguing Task Only Occurs at 75% MVC

To investigate the effects of our FT on muscle activity, we also evaluated EMG activity from two muscles during FT using a 2 × 3 RM-ANOVA with within-subject factors of muscle (FCR, ECR) and force level (5%, 50%, 75% MVC; Figure 2b). This revealed a significant main effect of muscle (*F*_1,59_ = 8.66, *p* = 0.006, η_p_^2^ = 0.23) and force level (*F*_2,58_ = 24.26, *p* < 0.001, η_p_^2^ = 0.46), as well as a significant muscle × force level interaction (*F*_2,58_ = 13.19, *p* < 0.001, η_p_^2^ = 0.31). Follow-up tests suggest that these differences are driven by significant differences at 75% MVC, suggesting that the magnitude of EMG activity only decreases during the highest FT.

#### 3.1.3. MVC Is Decreased After Both 50% and 75% MVC Force Levels

**Figure 3:**
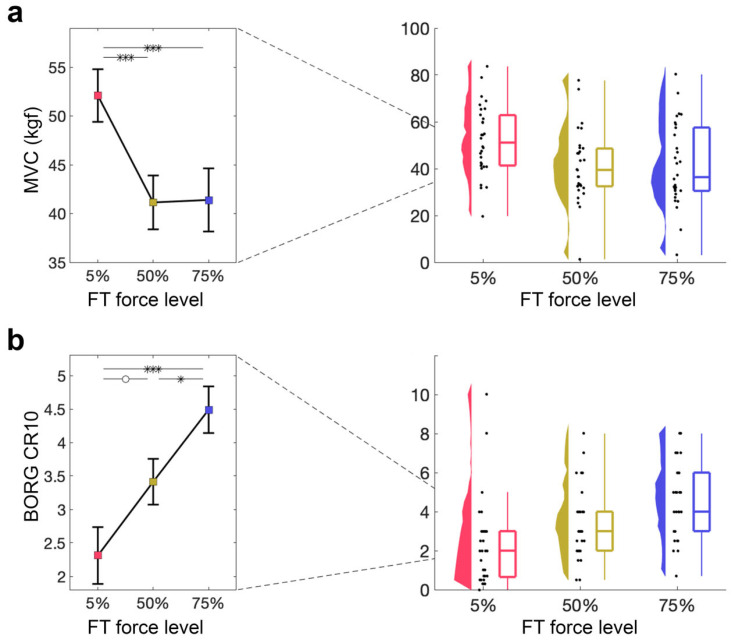
Indirect measures of fatigue during FT. (**a**) MVC directly after the FT for 5%, 50%, 75%. Mean and standard error across subjects are shown (**left**), as well as single-subject data, distribution, and boxplot (**right**). (**b**) Perceived exertion quantified using the BORG CR10 after the FT for 5%, 50%, 75%. Mean and standard error across subjects are shown (**left**), as well as single-subject data, distribution, and boxplot (**right**). ° *p* > 0.05, * *p* < 0.05, ** *p* < 0.01, *** *p* < 0.001.

Next, we examined how the ability to produce maximal contractions is affected by the FT, as quantified by the MVC performed directly after the FT. A 1 × 3 RM-ANOVA with a within-subjects factor force level (5%, 50%, 75% MVC) demonstrated a significant main effect of force level (5% MVC: *M* = 52.09, *SD* = 14.77; 50% MVC: *M* = 41.14, *SD* = 15.13; 75% MVC: *M* = 41.40, *SD* = 17.72; *F*2,58 = 13.89, *p* < 0.001, η_p_^2^ = 0.32; Figure 3a). Follow-up *t*-tests revealed significant differences between 5% and 50% MVC (*t*29 = 4.62, *p*bonf < 0.001, *d* = 0.69), as well as between 5% and 75% MVC (*t*29 = 4.51, *p*bonf < 0.001, *d* = 0.67), but not between 50% and 75% MVC (*p*bonf > 0.1), suggesting that 50% and 75% force levels similarly affect the MVC.

#### 3.1.4. Perceived Exertion Increases with Force Level of FT

Lastly, we examined how perceived exertion, as indexed by the BORG CR10 obtained at baseline and after the FT, was affected by different force levels during the FT. A 1 × 3 RM-ANOVA with a within-subjects factor of force level (5%, 50%, 75% MVC) showed a significant main effect of force level on BORG CR10 (5% MVC: *M* = 2.57, *SD* = 2.65; 50% MVC: *M* = 3.50, *SD* = 1.87; 75% MVC: *M* = 4.51, *SD* = 1.85; *F*2,58 = 12.79, *p* < 0.001, η_p_^2^ = 0.31; Figure 3b). Follow-up *t*-tests revealed significant differences between 5% and 75% MVC (*t*29 = −5.06, *p*bonf < 0.001, *d* = −0.90), as well as between 50% and 75% MVC (*t*29 = −2.62, *p*bonf = 0.033, *d* = −0.47), with a non-significant difference between 5% and 50% MVC (*t*29 = −2.43, *p*bonf = 0.054, *d* = −0.43), suggesting that perceived exertion increases with increasing FT force level.

#### 3.1.5. Relationship Between Fatigue Measures

To examine the associations between different fatigue metrics, we conducted correlation analyses using data from the 75% force condition. This condition was chosen because minimal fatigue is expected at 5% force, resulting in limited variability, whereas the 75% level represents the maximal fatigue condition in this study, where individual differences are most likely to be detectable and meaningful. The slope of force across contractions during the fatiguing task was significantly associated with the slope of EMG activity across contractions in both the FCR (*r*(28) = 0.647, *p* < 0.001) and ECR (*r*(28) = 0.449, *p* = 0.013) muscles. Additionally, the slope of force was negatively correlated with the MVC measured after the fatiguing task (*r*(28) = −0.446, *p* = 0.014). EMG magnitudes of the FCR and ECR muscles were also strongly correlated with each other (*r*(28) = 0.708, *p* < 0.001). In contrast, Spearman correlations did not reveal any significant associations between the BORG scale and other fatigue measures (all *p*s > 0.1).

### 3.2. Force Levels During the Fatiguing Task Affect the Magnitude of EMG Activity, but Not Kinematics, During the Assessment Task

Next, we examined whether the three different force levels (5%, 50%, 75% MVC) of the FT affected performance and muscle activity during AT for both the right (fatigued) and left (unfatigued) hand separately.

#### 3.2.1. No Significant Effect of Fatigue on Number of Target Hits

**Figure 4:**
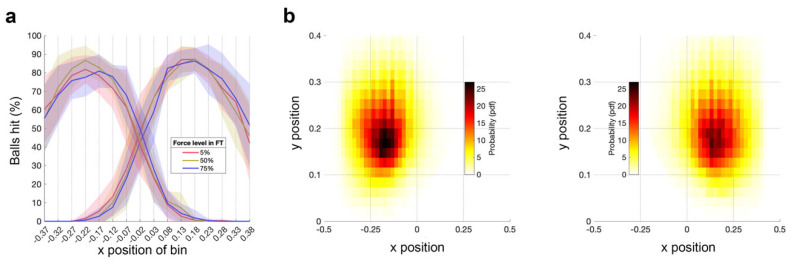
Ball hits and space covered per hand during the AT. (**a**) Ball hits per bin for the left (unfatigued) hand and the right (fatigued) hand during the AT performed after the FT for 5%, 50%, 75%. The solid line represents the mean across subjects; shaded area represents the standard error across subjects. (**b**) Heat map of the space covered by the left (unfatigued) hand and the right (fatigued) hand during the AT. Data are averaged across subjects and force levels of FT for 5%, 50%, 75%.

Figure 4a shows the hits per bin during the AT for the three FT force levels. A 2 × 3 RM-ANOVA with a within-subject factor of hand (right [fatigued], left [unfatigued]) and force level (5%, 50%, 75% MVC) demonstrated no significant main effect of hand or force level, and no significant hand × force level interaction (all *p*s > 0.1). The distribution of hit targets (Figure 4a) demonstrates the absence of ceiling or floor effects, which shows that the AT has sufficient dynamic range to capture potential fatigue-related changes.

#### 3.2.2. No Significant Effect of Fatigue on Space Covered

Next, we examined how the space covered during the AT by the right (fatigued) and left (unfatigued) hand is affected by different force levels during the preceding FT. Figure 4b shows the grand average heatmaps per hand. There were no significant differences between conditions for either hand.

#### 3.2.3. No Significant Effect of Fatigue on Acceleration and Velocity in AT

**Figure 5:**
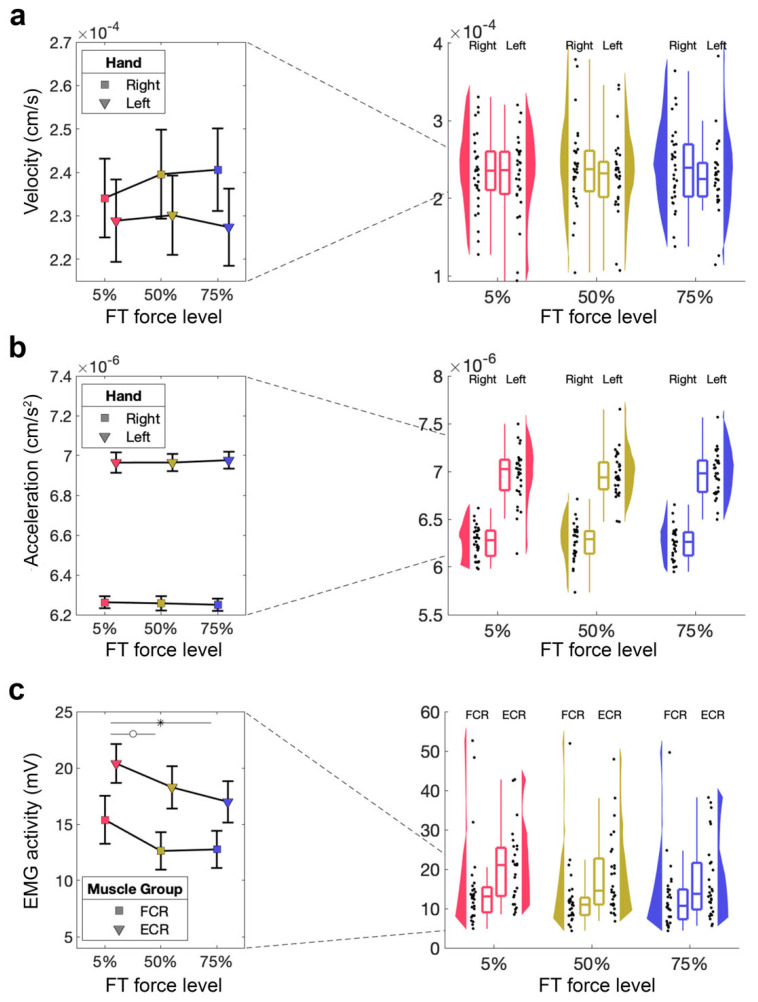
Kinematic measures and EMG activity during the AT. (**a**) Velocity of the fatigued (**right**) hand during the AT performed after the FT for 5%, 50%, 75%. Mean and standard error across subjects are shown (**left**), as well as single-subject data, distribution, and boxplot (**right**). (**b**) Acceleration of the fatigued (**right**) hand during the AT performed after the FT for 5%, 50%, 75%. Mean and standard error across subjects are shown (**left**), as well as single-subject data, distribution, and boxplot (**right**). (**c**) EMG activity of the fatigued (**right**) hand during the AT performed after the FT for 5%, 50%, 75%. Mean and standard error across subjects shown (**left**), as well as single-subject data, distribution, and boxplot (**right**). ° *p* > 0.05, * *p* < 0.05.

We then assessed if acceleration and velocity of the right (fatigued) and left (unfatigued) hand were affected by different FT force levels, using two separate 2 × 3 RM-ANOVA tests with the within-subject factors of hand (right [fatigued], left [unfatigued]) and force level (5%, 50%, 75% MVC). We found no significant main effect of hand or force level, and no significant hand × force level interaction on velocity (all *p*s > 0.1, Figure 5a). In terms of acceleration, we found a significant main effect of hand with higher acceleration for the left hand (*F*_1,29_ = 433.39, *p* < 0.001, η_p_^2^ = 0.88), but no significant main effect of force level (*p* > 0.1), and no significant hand × force level interaction (*p* > 0.1, Figure 5b).

#### 3.2.4. Assessment Task—Magnitude of EMG Activity

Finally, we wanted to test whether our fatiguing task made a significant difference to muscle activity in the AT, in the absence of behavioural effects. We therefore performed 2 × 3 RM-ANOVA tests with the within-subject factor of muscle (FCR, ECR) and force level (5%, 50%, 75% MVC) for each hand separately. For the right (fatigued) hand, this showed a significant main effect of force level (*F*_2,54_ = 4.98, *p* = 0.010, η_p_^2^ = 0.16), a non-significant difference for the main effect muscle (*F*_1,27_ = 4.09, *p* = 0.053, η_p_^2^ = 0.13), but no significant muscle × force level interaction (*p* > 0.1, Figure 5c). The follow-up *t*-test did not reveal a significant difference in EMG activity during the AT for 5% MVC compared to 50% MVC (*t*_27_ = 2.42, *p*_bonf_ = 0.059, *d* = 0.25) but a significant difference between 5% MVC and 75% MVC (*t*_27_ = 2.97, *p*_bonf_ = 0.013, *d* = 0.31), whereby 50% MVC was not significantly different from 75% MVC (*t*_27_ = 0.57, *p*_bonf_ = 1.00, *d* = 0.06). There was no significant main effect of force level or a significant muscle × force level interaction (both *p*s > 0.1) for the left (unfatigued) hand.

## 4. Discussion

In this study, we investigated the effects of unilateral handgrip fatigue on bilateral arm movement quality. We used multiple methods to assess (1) whether the FT protocol induced muscle fatigue, creating the necessary conditions for testing our hypotheses, and (2) how this fatigue affected movement quality in the bilateral task (AT).

### 4.1. Our Fatiguing Task Induced Fatigue Across Multiple Outcome Measures

To establish whether our FT induced fatigue in the target muscles (FCR and ECR), we used several metrics sensitive to detecting fatigue. We showed that the higher FT force levels led to a decline in real-time force output [29], changes in the magnitude of EMG activity [30], a decline in post-exercise MVC [1], and an increase in perceived exertion [31]. Collectively, significant changes were observed across all four metrics, and increasing force levels confirmed that muscle fatigue occurred with the FT protocol.

While force performance data showed significant differences across all force levels, EMG activity did not significantly differ between 5% and 50% MVC, only decreasing at 75% MVC. This may reflect compensation due to emergent handgrip fatigue at moderate force levels, likely mediated by increased muscle fibre recruitment [30]. In the 75% MVC condition, this compensation seemed to fail most in the ECR compared to the FCR. Post-exercise MVC showed a comparable decline for both 50% and 75% MVC, with no significant difference between the two conditions. When comparing the force data during the FT with the force in post-FT MVC, we see that higher force level exercise seems to affect repeated submaximal contraction performance more dramatically than maximum post-exercise contraction performance. This might be influenced by motivation, as participants may have felt more relief after completing the 75% MVC condition, thus improving subsequent MVC efforts. However, the self-reported exertion showed a more linear increase with force level. It is important to note that multiple experimental fatigue protocols exist. Although the protocol employed in the present study is well established and widely used, different protocols may induce fatigue through distinct physiological mechanisms and could therefore yield different outcomes. Future work should explore whether alternative fatigue-induction methods produce comparable or divergent effects.

In summary, our findings show that muscle fatigue was induced in both experimental conditions compared to the control, with a scaling effect among the 5%, 50%, and 75% MVC.

### 4.2. Handgrip FT Elicits Sustained Ipsilateral Electromyographical Effect Throughout AT

Analysis of EMG signal magnitude from the right arm during the AT shows that FT at both 50% and 75% MVC leads to a reduction in ipsilateral EMG activity compared to the control (5% MVC). This indicates that the implemented fatigue protocol shows a sustained fatiguing effect during the AT. Interestingly, the difference between the ECR and FCR muscles seen during the 75% MVC FT largely disappears in the subsequent AT. However, it is important to note that: (1) the continuous EMG measurements during the AT, compared to discrete contraction-based measurements during the FT, make a direct comparison challenging, and (2) comparing EMG activity between FT and AT is non-specific due to the differences in movement types between the two tasks.

### 4.3. Ipsilateral Arm Movement Quality in An Object-Hit Task Remains Unimpeded by Dominant Handgrip Fatigue

While participants showed signs of fatigue in their right arm, as indicated by EMG during the AT, this did not significantly affect hits, space covered, velocity, or acceleration of the fatigued (ipsilateral) arm. Contrary to our expectations, sustained unilateral handgrip fatigue did not impair ipsilateral arm movement quality in the bilateral object-hit task performed on the Kinarm end-point robot. This is likely due to the handgrip FT not being kinematically specific to the subsequent AT. Although handgrip exercises tend to elicit activity in most muscles of the upper extremity, the muscle groups involved in our FT and AT differ. The lack of performance deterioration of the fatigued (ipsilateral, right) arm in the AT explains why no spillover effect from the unfatigued (contralateral, left) arm was observed—there was no need for the unfatigued arm to support the fatigued homologue.

### 4.4. Contralateral Higher-Level Arm Movement Quality Is Unchanged Following Dominant Handgrip Fatigue

Past experiments from our group have shown that unilateral handgrip contractions at 50% MVC improve motor response latency in the contralateral, unfatigued, task-specific homologue and modulate cortical GABA-related activity. We aimed to test whether these effects translate into measurable changes in non-specific higher-level movement quality. However, neither the primary outcome (number of balls hit) nor the secondary outcomes (space covered, velocity, and acceleration during object-hit task) showed significant changes in the contralateral, unfatigued arm. Thus, our data suggest that unilateral handgrip fatigue does not affect non-specific higher-level movement quality of the contralateral homologue. The effects observed in our previously button-press task [3] did not translate to the object-hit task. Given that our AT involved motor activity in areas adjacent to but not specific to the FT (e.g., upper arm and shoulders), these findings should be interpreted within this context. Task-specific contralateral effects may be observed if the FT is more similar to the AT, as seen in our previous studies. The identical elements theory from motor learning suggests that greater transfer occurs when the training and assessment tasks share more common elements [32,33,34]. In the context of fatigue, this implies that the effects of fatigue would be more pronounced when the FT and AT are similar, and weaker when they are less similar. However, in daily life, FT and AT often differ, which may limit the ecological validity of using identical tasks. Therefore, we propose that exploring a broader range of FT and AT combinations similar to Schoenfeld et al. [35] is important, as this could reveal diverse patterns of motor fatigue, adaptation, and compensation. We believe that exploring a variety of FT and AT combinations is essential for fully understanding the effects of unilateral fatigue on bimanual motor performance.

### 4.5. Clinical Implications

Fatigue is common in patients with unilateral motor impairment, such as stroke survivors, and is one of the most frequently reported symptoms [36]. Post-stroke fatigue is thought to result from impaired corticomotor excitability [36], distinguishing it from physiological muscle fatigue, although some characteristics overlap. A decrease in corticomotor drive leads to muscle disuse, atrophy (especially type II fibres), reduced voluntary contraction, and impaired motor unit synchronisation [37], effects also seen in healthy individuals after inactivity [38]. Understanding how neural dynamics shape the adaptation to unilateral fatigue is both scientifically and clinically important.

While our findings did not show measurable improvements in contralateral arm movement quality, the lack of negative effects on the unfatigued arm is promising for clinical applications. Our results suggest that unilateral handgrip fatigue training does not impair contralateral arm performance, which could be useful for strength training and neuromodulatory benefits in rehabilitation. Research shows that high-intensity interval exercise promotes systemic benefits, such as increased brain-derived neurotrophic factor (BDNF) [39] and other factors supporting neuroplasticity. Although our study was conducted in healthy volunteers and assessed movement quality on the same day as the fatigue task, these findings suggest that unilateral fatigue tasks could be incorporated into rehabilitation without affecting the unaffected limb. For example, in patients with unilateral motor impairment, such as stroke survivors, managing fatigue in the affected limb while maintaining functionality in the unaffected limb could optimise rehabilitation efforts. Additionally, in cases where the unaffected limb also experiences some impairment (albeit less severe), it is important to ensure that training the unaffected limb does not exacerbate the deficits in the more affected arm. Similarly, in sports like tennis or baseball, where athletes rely heavily on a dominant arm for powerful strokes, the ability to train and fatigue one arm while maintaining performance in the non-dominant arm could enhance overall performance and help prevent injury. Further studies are needed to explore how such protocols could be refined and applied in clinical settings to enhance motor recovery.

### 4.6. Conclusions

This study shows that unilateral handgrip fatigue at moderate to high intensities (50% and 75% MVC) does not impair bilateral arm movement quality in an object-hit task, despite inducing significant muscle fatigue. The fatigue protocol elicited robust effects, as evidenced by significant changes in both direct (force output, EMG activity) and indirect (post-exercise MVC, BORG ratings of perceived exertion) measures. However, no significant changes were observed in movement performance or quality in either the fatigued (ipsilateral) or unfatigued (contralateral) arm during the object-hit task using the Kinarm robot, despite lower EMG activity in the muscles involved in the fatiguing task. These findings suggest that while unilateral handgrip fatigue affects muscle activity of handgrip muscles, it may not translate into changes in bilateral arm movement quality, possibly due to the nature of the movements involved and compensation mechanisms that preserve overall movement performance. Previously reported improvements on contralateral response latency in a button-press task were not found to translate into advanced arm movement quality benefits.

## Data Availability

The raw data that support the findings of this study are available at https://doi.org/10.60964/rnd-rtyw-9w81. The code for the FT task is available at: https://github.com/cathazi/task-Fatigue.git. The code for the analysis is available at: https://github.com/cathazi/Knorz_2025_Fatigue.git.

## Abbreviations

The following abbreviations are used in this manuscript:

MVC	Maximum voluntary contraction force
KINARM	Kinesiological instrument for normal and altered reaching movement
fMRI	Functional magnetic resonance imaging
MRSI	Magnetic resonance spectroscopic imaging
GABA	Gamma-aminobutyric acid
SMA	Supplementary motor area
IHI	Interhemispheric inhibition
TMS	Transcranial magnetic stimulation
SICI	Short interval intracortical inhibition
APB	Abductor pollicis brevis
MEP	Motor evoked potential
IQR	Interquartile range
CUREC	Oxford Central University Research Ethics Committee
AT	Assessment Task
FT	Fatiguing Task
MVC 3×	3 cued contractions at MVC, each of 3 s duration with 30 s rest in between
AGQ-S	2 × 2 Achievement Goals Questionnaire for Sport
EHI	Edinburgh Handedness Inventory
GPAQ	World Health Organization Global Physical Activity Questionnaire
EMG	Electromyography
FCR	Flexor carpi radialis
ECR	Extensor carpi radialis longus
TMSi	Twente Medical Systems International
FWHM	Full width at half maximum
AUC	Area under the curve
RM-ANOVA	Repeated-measures analysis of variance
M	Mean
SD	Standard deviation
BNDF	Brain-derived neurotrophic factor
